# Empowering the future of evidence‐based healthcare: The Cochrane Early Career Professionals Network

**DOI:** 10.1002/cesm.70006

**Published:** 2024-10-22

**Authors:** Ana Beatriz Pizarro, Santiago Castiello‐de Obeso, Ahmad Sofi‐Mahmudi, Robin Vernooij, Elpida Vounzoulaki, Chris Champion

**Affiliations:** ^1^ Clinical Research Center, Fundación Valle del Lili Cali Colombia; ^2^ Cochrane Central Executive Team London UK; ^3^ Wu Tsai Institute Yale University New Haven Connecticut USA; ^4^ Department of Health Research Methods, Evidence and Impact McMaster University Hamilton Ontario Canada; ^5^ Department of Nephrology & Hypertension University Medical Center Utrecht Utrecht The Netherlands; ^6^ Julius Center for Health Sciences and Primary Care, University Medical Center Utrecht Utrecht University Utrecht The Netherlands; ^7^ Leicester Real World Evidence Unit, Diabetes Research Centre University of Leicester Leicester UK; ^8^ MAGIC Evidence Ecosystem Foundation Oslo Norway

## 1 INTRODUCTION

The Cochrane Early Career Professionals Network (ECPN) is a diverse global network of emerging volunteer health professionals committed to advancing evidence‐based healthcare worldwide [1]. Established in September 2019 by Robin Vernooij and Chris Champion, the ECPN was inspired by similar early career researcher groups in scientific associations. Cochrane received many inquiries from young researchers eager to engage with the activities, highlighting a growing demand for such a network.

Cochrane's contributors come from varied backgrounds and cultures, including researchers, health sciences students, language translators, and other volunteers, all united in their commitment to supporting global healthcare initiatives [2]. In line with Cochrane's mission to highlight the contributions of the next generation, Cochrane launched the “30 Under 30” initiative, inviting 30 young researchers with diverse professional backgrounds such as nurses, doctors, dentists, physiotherapists, biologists, psychologists, and journalists to join the series and tell their Cochrane Story. To build on the success of this initiative, Vernooij proposed formalizing this group into a network, which was well received as the idea aligned perfectly with the Cochrane membership strategy. Consequently, all participants in the “30 Under 30” series were invited to become the first members of the ECPN [2]. The ECPN was due to be launched in Santiago, Chile at the 2019 Cochrane Colloquium, themed “Embracing Diversity,” but due to the cancellation of the event, it was ultimately launched online later in 2019.

The ECPN now plays a crucial role in promoting professional development, international networking, collaboration, and leadership within the Cochrane community, ensuring that the voices of early career professionals (ECP) are heard, and their potential maximized [1]. The objective of this commentary is to provide a comprehensive overview of the ECPN in Cochrane, with the aim of raising awareness and enhancing the visibility of the network within the Cochrane and scientific community.

## 2 MISSION AND OBJECTIVES

The ECPN's mission is to enhance its members' knowledge, skills, and expertise while fostering leadership development and active participation in shaping Cochrane's future. Four core objectives guide its activities:

- International Networking: Facilitating the exchange of knowledge, experiences, and ideas, promoting collaboration, and fostering research partnerships.
- Trainee Representation: Ensuring the active involvement of early career professionals in Cochrane's decision‐making processes and strategic direction.
- Active Patient Involvement: Exploring innovative approaches to engaging patients, particularly young people, in research and knowledge translation activities, with the support of the Cochrane Consumer Executive.
- Knowledge Translation: Investigating strategies to improve the dissemination and impact of Cochrane reviews to improve healthcare decision‐making.

## 3 PRESENT INITIATIVES

**Figure 1:**
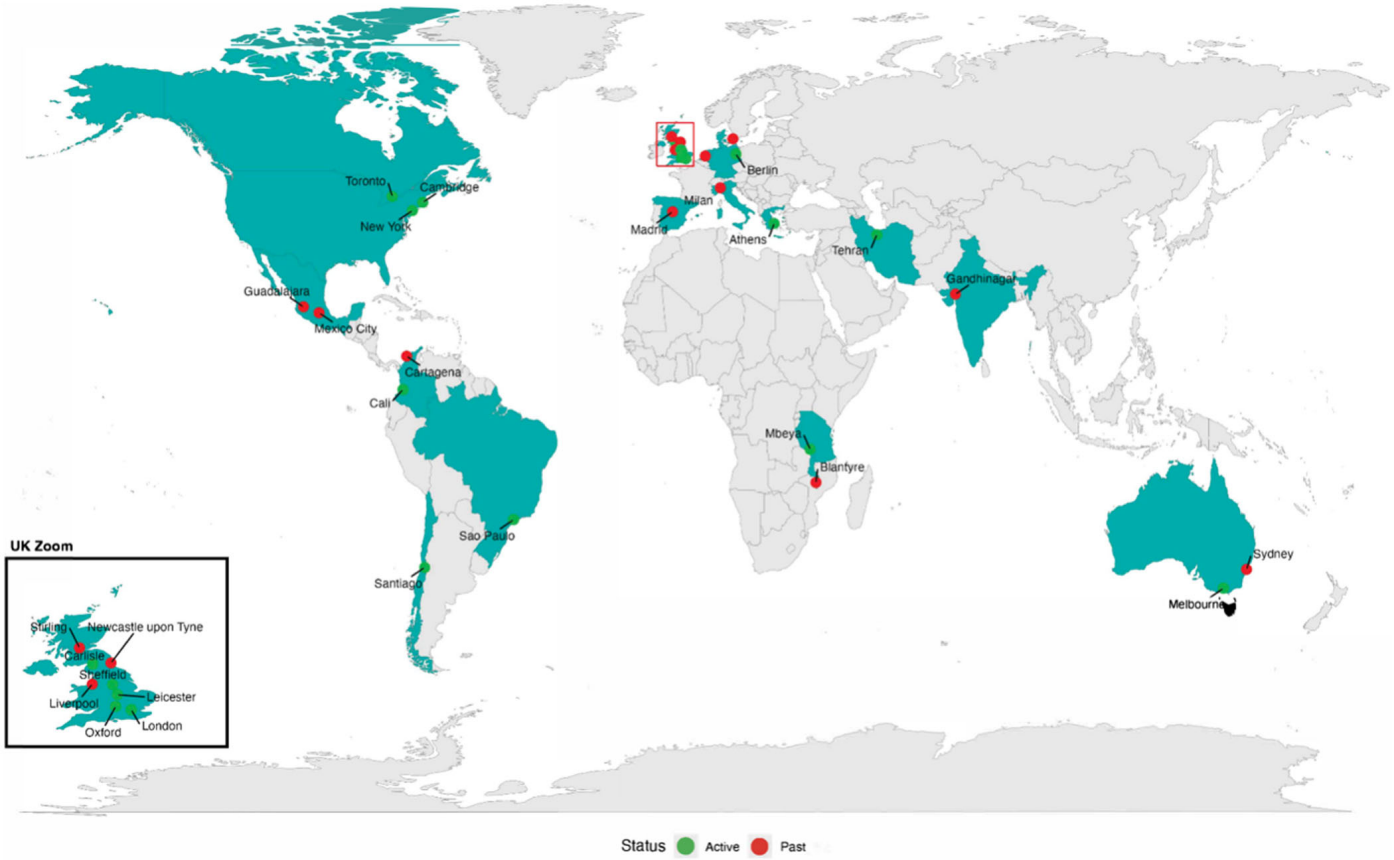
Global geographical distribution of active (in green) and past (in red) members of the Early Career Professionals Network (ECPN). The map illustrates the presence of ECPN members in regions across North and South America, Europe, Africa, Asia, and Australia.

The ECPN is focused on expanding our international network and fostering closer collaboration with other groups, such as the Campbell Collaboration, the Joanna Briggs Institute, Sense about Science, and the Cochrane US mentoring program [3]. We actively explore new ways to involve individuals at the early stages of their careers in research, including students, and young professionals in our research and projects, ensuring their perspectives are integrated into our work. Additionally, we are working on innovative approaches to knowledge translation, aiming to make Cochrane reviews more accessible and impactful. The network consists of a steering group, led by a Chair, that supports the early career professionals' community. We currently have 15 active members, and over the 5 years since the network's founding, a total of 50 members have participated. Membership rotates every 2 years through an application process followed by a scoring and consensus vote among current members. Additionally, the Chair is elected biennially. Our members represent a global presence, spanning all continents—from Chile to Australia, including the United States, Europe, and several African countries. The geographical distribution of active and past members of the ECPN is represented in Figure 1. This map visually highlights the global presence of ECPN members, with clusters concentrated in regions such as Europe, South Asia, Latin America, and North America.

## 4 ACTIVITIES AND CONTRIBUTIONS

The ECPN's diverse activities align with its core objectives. These include:

- Representation in governance and management boards within Cochrane: Ana Beatriz Pizarro was the first ECP to join the Cochrane Editorial Board and the Consumer Executive, while Santiago Castiello‐de Obeso and Ahmad Sofi‐Mahmudi represented us on the Cochrane Council.
- Journal Clubs: Critical appraisal of published research to enhance methodological understanding.
- Cochrane ECPN Podcast: “Conversations with Cochrane”, dissemination of knowledge and insights to a broader audience.
- Newsletter: Communication of news, events, and opportunities to members.
- Lecture Series: Educational programs focused on systematic reviews and research methods.
- Educational Materials: Development of resources tailored to the needs of early career researchers, such as the Evidence‐Based Nuggets initiative, containing short summaries of recent and/or interesting Cochrane reviews.
- Global Presence Projects: Expansion of ECPN's reach and impact through lectures and workshops.
- Online Meetups: Networking and knowledge‐sharing opportunities in a virtual setting.
- Partnering with the Cochrane Consumer Network: Provide plain language summaries and evidence‐based resources to patients and caregivers ensuring that high‐quality evidence was accessible and tailored to their needs.
- Collaborative Research: Co‐authorship and publication of impactful research studies [4].
- International Conferences: Active participation and presentation of research findings at Cochrane's annual conference and the Global Evidence Summit.

## 5 LOOKING TO THE FUTURE

The ECPN has already demonstrated immense value in encouraging the next generation of evidence‐based healthcare (EBHC) experts. With a focus on international collaboration, consumer involvement, and knowledge translation activities, the ECPN provides the platform to engage in the evolving challenges of EBHC. As the ECPN continues to grow and mature, its potential to influence the field is immense.

Since its inception, the network has made significant strides in supporting early career professionals. With more than 1.6k followers on X @CochraneECP, twenty bi‐monthly newsletters since 2020, the network has successfully hosted more than 15 journal clubs, podcasts, and lecture series, attracting participants from around the world. These newsletters have been a valuable resource for keeping members informed and engaged, and its projects aimed at increasing global presence have enhanced the visibility and influence of the ECPN within the Cochrane community; we also promote the use of Cochrane platforms such as Cochrane Crowd, Cochrane Engage, and the Cochrane/Wikipedia project.

## 6 FUTURE DIRECTIONS

Looking ahead, the ECPN aims to strengthen its international network further and enhance professional development opportunities for its members. Potential future directions include:

1. Expanding its focus to incorporate methodological advancements in network meta‐analysis, living systematic reviews, and artificial intelligence applications, the ECPN will support the network by facilitating access to training workshops, webinars, and collaborations with established experts in these areas. Additionally, we aim to partner with Cochrane's Methods Groups, the Cochrane and other expert bodies to ensure that our members can participate in ongoing methodological advancements and apply these techniques in their research.
2. Increasing collaboration with other early career professional networks and societies in related fields.
3. Establishing formal mentorship programs or partnering with the Cochrane US health equity mentoring program or the international mobility program [5], to support the development of early career researchers, with structured evaluations based on participant feedback, career progression, and research outcomes.
4. Exploring the role of social media projects in disseminating evidence‐based information to the public.
5. Introducing new initiatives to provide early career professionals with more leadership opportunities and avenues for active involvement in Cochrane's mission and vision, evaluation metrics will include leadership outcomes, increased participation in Cochrane activities, and feedback from participants.
6. Continuing to explore ways to improve student involvement and knowledge translation, ensuring that the work of the ECPN remains relevant and impactful.

By embracing these opportunities, the ECPN can further solidify its position as the leading network for ECP in EBHC and contribute to its ongoing evolution, every ECP in the Cochrane community is warmly invited to join this network, which we now consider our extended family.

## 7 CONCLUSION

The Cochrane ECPN is dedicated to supporting the next generation of health professionals and researchers in engaging with EBHC. Through the creation of an international community of volunteers, we have fostered a strong network that supports one another unconditionally. This is perhaps our most valuable achievement—building a community that thrives on collective support and collaboration. Our members remain actively involved in knowledge translation and dissemination activities, despite the challenges of limited funding and capacity.

By providing a platform for networking, professional development, and active participation in Cochrane's strategic initiatives, we aim to empower early career professionals to make meaningful contributions to the health research field. Our past accomplishments, current initiatives, and future aspirations reflect our commitment to enhancing the skills and expertise of our members and advancing Cochrane's mission, vision, and the Scientific Strategy 2025‐2023.

## AUTHOR CONTRIBUTIONS

**Ana Beatriz Pizarro**: Conceptualization, writing—original draft, reviewing, and editing. **Elpida Vounzoulaki**: Writing—review & editing. **Santiago Castiello‐de Obeso**: Writing—review & editing. **Ahmad Sofi‐Mahmudi**: Writing—review & editing, **Robin Vernooij**: Writing—review & editing. **Chris Champion**: Writing—review & editing.

## CONFLICT OF INTEREST STATEMENT

The authors declare no conflict of interest.

## PEER REVIEW

The peer review history for this article is available at https://www.webofscience.com/api/gateway/wos/peer-review/10.1002/cesm.70006.

## Data Availability

Data sharing not applicable to this article as no data sets were generated or analyzed during the current study.
